# First molecular screening of deafness in the Altai Republic population

**DOI:** 10.1186/1471-2350-6-12

**Published:** 2005-03-24

**Authors:** Olga Posukh, Nathalie Pallares-Ruiz, Vera Tadinova, Ludmila Osipova, Mireille Claustres, Anne-Françoise Roux

**Affiliations:** 1 Institute of Cytology and Genetics, Siberian Branch of Russian Academy of Sciences, Novosibirsk, Russia; 2 Laboratoire de Génétique Moléculaire, Institut Universitaire de Recherche Clinique, CHU Montpellier, Montpellier, France; 3 Republican Altai Children's Hospital, Gorno-Altaisk, Republic Altai, Russia

## Abstract

**Background:**

We studied the molecular basis of NSHL in Republic of Altai (South Siberia, Russia). The Altaians are the indigenous Asian population of the Altai Mountain region considered as a melting-pot and a dispersion center for world-wide human expansions in the past.

**Methods:**

A total of 76 patients of Altaian, Russian or mixed ethnicity and 130 Altaian controls were analyzed by PCR-DHPLC and sequencing in the *GJB2 *gene. The *GJB6 *deletion and the common non-syndromic deafness-causing mitochondrial mutations were also tested when appropriate.

**Results:**

8.3% of the Altaian chromosomes were carrying *GJB2 *mutations versus 46.9% of the Russian chromosomes. The 235delC mutation was predominant among Altaians, whereas the 35delG mutation was most prevalent among Russian patients.

**Conclusion:**

We found an Asian-specific *GJB2 *diversity among Altaians, and different *GJB2 *contribution for deafness in the Altaian and Russian patients. The high carrier frequency of 235delC in Altaians (4.6%) is probably defined by gene drift/founder effect in a particular group. The question whether the Altai region could be one of founder sources for the 235delC mutation widespread in Asia is open.

## Background

Non-syndromic hearing loss (NSHL) accounts for approximately 80% of cases of hereditary deafness and variety of genes are involved in NSHL [1]. Mutations in the *GJB2 *gene, encoding the connexin 26 gap-junction protein, account for a significant proportion of NSHL [2]. To date, the substantial contribution of several nuclear genes [3,4] and the pathogenic mitochondrial mutations in NSHL was established in some populations [5,6]. Different population distributions of more than 70 *GJB2 *mutations have been described and prevalence of some *GJB2 *mutations is known to depend on population ethnic origin [2,7]. The high frequencies of the 35delG mutation in the Caucasians, the 167delT in Ashkenazi Jews, and the 235delC mutation among east Asian populations have been shown to be the results of founder effects [8-12]. The underlying mechanisms accounting for the high prevalence of certain founder *GJB2 *alleles are unknown. The data concerning the molecular basis of deafness in the populations of Russia are scarce and mostly limited to the European part of Russia and to the screening of the sole mutation 35delG [13-16]. Such data are yet unknown for Siberia (that is an expansive Asian part of Russia) populated by different ethnic groups.

The investigation presented here was performed on individuals with HI and subjects with normal hearing who are living in the Republic of Altai, situated in South Siberia and bordering Mongolia, China and Kazakhstan. Because of location, the Altai have been a particular region of great evolutionary importance. The archaeological, anthropological, and recent genetic data, mainly based on Y-chromosome and mitochondrial DNA studies [17,18] suggest that for thousand of years the Altai-Sayan Mountains with surrounding territories have been a zone of the first contacts between paleo-Caucasoids and Mongoloids; repeatedly this region was a peculiar "hybrid" area closely connected with Central Asia, and a source of subsequent human migrations.

Indigenous inhabitants of the Altai Republic are the Altaians originated from several ancient Turkic-speaking tribes [19]. The Altaians mainly belong to the Central Asian type of the North Asian race. The Altai Republic population accounts for about 200,000 including Altaians, Russians, Kazakhs and other nationalities. The total number of Altaians is about 60,000. The rural population constitutes 3/4 of the Altai Republic people whereas approximately 50,000 persons live in the city of Gorno-Altaisk. The Altaians currently living in small settlements in the distant regions of the Altai Republic have mainly retained their native language, ethnic identity, and traditional marriage structure (patrilineal clan exogamy) [20,21]. On the contrary, the population of the town Gorno-Altaisk is characterized by higher rates of interethnic marriages among Altaians, Russians and other ethnicities. As a whole, the Altaian population was shown to be genetic heterogeneous resulted from its territorial subdivision and the specific Altaian clan compositions in different localities [20-22]. Recently, the first epidemiological data in Altai Republic revealed the spectrum of hereditary pathology in the Altai Republic population with different prevalence rates among the urban and the rural populations, and among main ethnic groups of population (Russians, Altaians, Kazakhs) [23].

We report here the results of a study based on patients presenting mainly prelingual deafness, with the mutation analysis of the *GJB2 *gene, the screening of the *GJB6*-D13S1830 deletion, and the study of five mitochondrial mutations involved in NSHI. Moreover, the *GJB2 *screening was performed in 130 unrelated control subjects of Altaian ethnicity.

## Methods

### Patients

This study was approved by the Altai Republic Ministry of Health. Data on individuals with hearing impairment were obtained from the Republican medical institutions, Association of Deaf People, Specialized School for Deaf Children of the Republic of Altai (the town Gorno-Altaisk), and local settlements on the territory of the Republic. We ascertained 76 patients with HI (37 males and 39 females) of different ethnic affiliation (Altaians, n = 40; Russians, n = 17; Kazakhs, n = 3; mixed or other ethnicity, n = 16) representing a total of 51 unrelated families. Their ages ranged from 3 to 80 years old (mean 30.2). Based on family histories, 19 patients were defined as sporadic cases, while 57 patients were issued from 32 unrelated families, presumably showing an autosomal recessive (20 families), autosomal dominant or maternal (8 families), and ambiguous (4 families) mode of inheritance. In addition to the patients, 58 DNA samples of family members with normal hearing were included. Informed consent was obtained from all adult participants and from parents of the children studied.

Bilateral (mainly symmetrical) and prelingual/early onset HL was defined in most of the patients. Among the 76 patients 44 could be otoscopically examined, and pure tone audiometry was performed in the special medical service (of the town Gorno-Altaisk). As the other patients were mostly living in the small settlements distant from the town Gorno-Altaisk, the data was collected from the local unspecialized medical services and by direct interview with the patients and their relatives. Among the clinically documented patients (n = 44), HL was severe to profound in 32 patients, moderate in 10, and mild in 2 patients. Sensorineural type of HI was precisely defined for 38 out of 76 patients, and the remaining patients presented mixed (conductive-sensorineural, n = 6) or uncertain type of HL. It should be noted that the chronic otitis media, widespread in Altaian population, was frequently registered in the medical histories of these patients. We have analyzed the cohort of patients with HI without the possibility of excluding cases of environmental exposures (infections, prenatal or postnatal ototoxicity etc) or unknown etiology.

### Controls

130 unrelated subjects of Altaian origin with no familial history of hearing problems were used as controls. Most of DNA samples were obtained from the Southern Altaian population of the Ust'-Kan administrative region of the Altai Republic and the others from Northern Altaians living in the Turochak administrative region. All DNA samples studied were anonymized.

### Mutation analysis

DNA was extracted from peripheral blood using standard protocols. *GJB2 *analysis was performed for the non-coding exon E1 and coding exon E2 and their flanking sequences by PCR-DHPLC as previously described [24]. Any abnormal profile observed by DHPLC was consequently analyzed by sequencing (ABI 310 sequencer, Applied Biosystems). The patients without any mutations in the coding exon E2 were analyzed in the non-coding exon E1 and tested for the mutation Δ(*GJB6*-D13S1830) that includes the deletion of most of the *GJB6 *gene [24]. The screening of five known mitochondrial mutations (m.A1555G, m.7445A>G, m.7472insC, m.7510T>C, m.7511T>C) was performed in the *GJB2 *negative patients that were compatible with a maternal inheritance of HI. The A1555G mutation was analyzed as previously described [5] whereas the four others were analyzed by direct sequencing (position 7321–7620 of the mtDNA).

### Statistical analysis

Differences between groups were analyzed by chi square statistics. *P*-values were considered to be significant when <0.05.

## Results

### *GJB2 *gene

**Figure 1:**
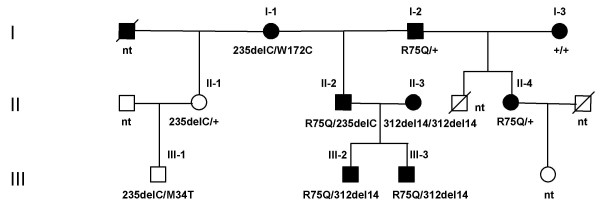
**Pedigree of family 11 presented with the *GJB2 *genotypes. **Analyses were performed for the numbered subjects. ''+'' denotes that no *GJB2 *mutations were detected; ''nt'' – not tested. Ethnic affiliation of the parents: I-1 was Altaian; I-2, I-3, father of II-1 and father of III-1 were Russians; II-3 was of mixed ethnicity (Russian/Tatar).

*GJB2 *mutations were detected in 23.7% of the patients (18 out of 76) (Table 1). We identified five different mutations, all of them lying in the coding exon: three known as recessive mutations (35delG, 235delC and 312del14), one as a dominant mutation (R75Q), the fifth mutation is a newly identified missense mutation, W172C. All the patients with *GJB2 *mutations were familial cases presenting prelingual profound HL except one patient, genotyped 35delG/235delC, who had moderate-severe HL. Mutations were present as homozygous or compound heterozygous state, with the exception of the R75Q that was identified as single in two members of family 11. Among all the families studied, family 11 deserves particular interest because dominant and pseudo-dominant deafness is observed with a complex segregation of different mutations, as shown on Fig. 1 (see in Discussion).

Table 2 shows the distribution of *GJB2 *sequence variations in the two predominant ethnic groups (Altaians and Russians) of patients as well as in Altaian controls. Of the 60 unrelated Altaian patient alleles, only 8.3% were carrying *GJB2 *mutations with the 235delC representing 6.67% of the alleles and the W172C, 1.67%. In contrast, 46.9% (15 out of 32) of the unrelated Russian patient chromosomes carried *GJB2 *mutations with the 35delG accounting for the most frequent mutation (34.4%). The other mutations 312del14, R75Q and 235delC were detected in 6.3%, 3.1% and 3.1% of the Russian alleles, respectively.

The polymorphism V27I and E114G were observed in Altaian patients as well as in Altaian controls (Tables 1 and 2). None of the Russian patients had both variants. Because the E114G variant was never identified as single in our study but also in others [25,26] we assumed that the E114G and V27I variants are in *cis*-configuration. The estimated allele frequencies of V27I and [V27I; E114G] are respectively 16.7% and 11.7% among 60 unrelated Altaian patient chromosomes, versus 13.1% and 6.5% in the Altaian controls (260 chromosomes). Furthermore, three other known sequence variations were identified in Altaian controls: the 235delC mutation (2.3%), the F191L variation with unknown pathogenic consequences (0.4%), and the non-pathogenic V153I variant (0.4%) as shown on Table 2. A new sequence variation, IVS1+27G>C, was identified in one Altaian patient with severe-profound HL who had genotype IVS1+27G>C / [V27I; E114G] (Table 1) and in his non-affected mother (genotyped IVS1+27G>C/+). The IVS1+27G>C variation was not previously described, but splice site prediction programs would suggest its non-pathogenic consequences [27]. Finally, the 765C>T polymorphism (referred to as SNP1 [9]) located in the 3'UTR of the *GJB2 *gene was analyzed in patients. All alleles carrying the 35delG mutation were associated with the 765T variant; that is in accordance with recent data on the French patients [24].

### Δ(*GJB6*-D13S1830) and mitochondrial mutations

Screening of the Δ(*GJB6*-D13S1830) and mitochondrial mutations m.1555A>G, m.7445A>G, m.7472insC, m.7510T>C, m.7511T>C was negative in patients who had no *GJB2 *mutations.

## Discussion

Specific *GJB2 *mutation spectrums and different *GJB2 *contributions for deafness were observed for the two main ethnic groups studied here.

### Prevalence of *GJB2 *mutations in Russian patients

*GJB2 *mutations are responsible for deafness in 54% (7 out of 13) of the independent Russian families tested and the 35delG mutation was carried by 34.4% of the Russian alleles. These findings correlate with earlier reported data concerning Russian subjects with HI [13-16] and confirm the main contribution of the 35delG mutation in deafness among Caucasian populations [28,29].

### *GJB2 *mutations with dominant and pseudo-dominant transmission

We studied the segregation of different *GJB2 *mutations in a three-generation family (family 11; Fig. 1). All affected members presented prelingual profound HL and were using sign language to communicate. The dominant mutation R75Q (224G>A) was found in five members, as the sole mutation or in *trans *of another mutation (235delC or 312del14). R75Q was previously identified as a non-syndromic dominant mutation [7] and as a syndromic mutation [30] in a four-generation Turkish family with autosomal dominant inherited HI and congenital diffuse palmoplantar keratoderma. The age of onset and progression of HL were variable among the affected members of this family [30]. It should be noted that a previously described dominant mutation located at the same codon 75 (R75W) was found to have a variable penetrance regarding skin disease symptoms (even to their absence) [31,32]. Preliminary dermatological examination performed in all individuals of family 11 carrying the R75Q mutation did not reveal any skin disease in patients I-2, II-2 and II-4 though mild manifestation of probable keratosis was detected in patients III-2 and III-3. However, additional investigations need to be performed for elucidation of non-syndromic and/or syndromic status of the R75Q mutation as other factors can modulate the expression of a particular phenotype. The new missense mutation W172C (516G>C) was identified in *trans *of the 235delC allele in the Altaian patient I-1. This patient had both parents and a brother with normal hearing but an affected sister (not shown). A G>A substitution at position 516, generating a stop codon at position 172 (W172X) had been previously described [7]; moreover, the tryptophan at position 172 is conserved in many species (human, mouse, rat, cow, sheep). These data tend to imply that this *GJB2 *sequence variation is a severe recessive mutation although *de novo *appearance of the W172C mutation cannot be excluded in this case.

### *GJB2 *contribution in Altaian patients and controls

This study has revealed an Asian-specific diversity and the prevalence of *GJB2 *sequence variations in the Altaians. High rates of the V27I and [V27I; E114G] alleles were detected in Altaian patients and Altaian controls with no significant differences between both groups (p > 0.05). These results, once more, confirmed that both sequence variations correspond to non-pathogenic polymorphisms and (that their high rates) represent a distinctive feature of particular Asian populations [10-12,25,33-36]. *GJB2 *gene contribution in deafness among Altaians (8.3%) is mainly defined by the recessive mutation 235delC (6.67%). This mutation is specific to some east Asian populations (Japan, China, Korea, Taiwan) with an allelic frequency up to 20% among deaf patients and a carrier frequency of 0% to 2.8% in normal populations and it represents the most prevalent *GBJ2 *pathogenic mutation as it accounts for up to 80% of the pathogenic alleles in patients [10-12,33-37].

The 235delC mutation, found in 4/60 unrelated chromosomes in Altaian patients, was also detected in 6/130 Altaian controls resulting in a carrier frequency of 4.6%, higher than in any other Asian populations. This high frequency could be attributed to the gene drift/founder effects in a certain subpopulation. The Altaian control group studied here consists mainly of the inhabitants of one administrative region of the Altai Republic. Earlier, it was shown that the marriage migrations among this Altaian subpopulation were, in their majority, restricted to the territory of this region [21].

Comparatively low *GJB2 *contribution among the Altaian patients (8.3%) could be explained by the fact that a few patients presented acquired hearing loss. Moreover, a specific marriage structure (patrilineal clan exogamy) practiced by the Altaians for a long time to avoid close inbreeding, could influence apparent scarcity of affected *GJB2 *homozygotes by restriction of random marriages of Altaians belonged to certain clans. Finally, some other deafness-causing genes, not considered in this study, could be involved in deafness among Altaians. The additional study of the *GJB2 *gene diversity in other Altaian groups will be informative for verification of the 235delC prevalence pattern among the Altaians.

Recent studies had established that the 235delC mutation among east Asian populations was derived from a common ancestral founder [10-12]. Yan et al. [12] roughly estimated the age of the 235delC mutation to be about 11500 years old and speculated that the 235delC mutation might have arisen in the Lake Baikal area and then spread to Mongolia, China, and Japan through subsequent migrations. Taking into account that the territories of South Siberia, including the Altai region and the Lake Baikal area were melting-pots and dispersion centers for world-wide human expansions in the past, we suggest that the Altai region could be one of the founder sources for the 235delC mutation widespread in Asia. To elucidate this hypothesis, the 235delC prevalence will be tested among indigenous people living in other regions of Altai and surrounding territories and SNP analyses will be performed to verify the common origin of the 235delC alleles.

## Conclusion

In this study we found an Asian-specific *GJB2 *diversity among the Altaians and different *GJB2 *contribution for deafness in the Altaian and Russian patients. Additional investigations remain necessary to elucidate all genetic origins of HL in Altaian patients. The high carrier frequency of the 235delC mutation in normal hearing Altaians (4.6%) is probably defined by gene drift/founder effect in a particular Altaian group. The question whether the Altai region could be one of the founder sources for the 235delC mutation widespread in Asia is open. Finally, we presented new evidence that the CX26 R75Q mutation, earlier described as syndromic mutation (autosomal dominant inherited hearing impairment with congenital diffuse palmoplantar keratoderma), could be associated with a high variable expression of the skin symptoms.

## Competing interests

The author(s) declare that they have no competing interests.

## Authors' contributions

VT and OP collected the family data and recruited patients and controls for the study; LP provided a part of control blood samples; OP and NPR carried out the molecular studies; AFR supervised the whole study in the laboratory of MC. AFR and OP wrote the manuscript. All authors read and approved the final version of the manuscript.

## Pre-publication history

The pre-publication history for this paper can be accessed here:



## Figures and Tables

**Table 1:** *GJB2 *sequence variations identified in patients

Genotype	Affected subjects (n = 76)	Ethnic affiliation
Mutations		
35delG / 35delG	7 (4 independent families)	Russians
235delC / 235delC	1	Altaian
312del14 / 312del14	1	Russian/Tatar
35delG / 235delC	2 (unrelated)	1 – Russian/Altaian, 1 – Russian
35delG / 312del14	1	Russian
R75Q^a ^/ 235delC	1	Altaian/Russian
R75Q / 312del14	2 (two sibs)	mixed ethnicity
R75Q/ +	2 (father and daughter)	Russians
235delC / W172C	1	Altaian
Total	18 (23.7%)	
Non-pathogenic variants		
V27I / +	14 (11 independent families)	13 Altaians, 1 Kazakh
(V27I; E114G) ^b^	7 (unrelated)	6 Altaians, 1 Altaian/Russian ^c^
IVS1+27G>C / [V27I; E114G]	1	Altaian
Total	22 (28.9%)	

^a ^dominant mutation; ^b ^*cis*-configuration of V27I and E114G was confirmed by family studies in 5/7 patients, ^c ^the [V27I; E114G] allele of this proband is known to be of Altaian origin (maternal branch).

**Table 2:** Frequency of the *GJB2 *sequence variations among patients and Altaian controls

Name	Nucleotide change	Number of unrelated chromosomes tested Frequency (95% CI)		
		
		Altaian patients^a ^n = 60	Altaian controls n = 260	Russian patients^b ^n = 32
235delC	-	4 0.067 (0.019–0.144)	6 0.023 (0.009–0.045)	1 0.031 (0–0.118)
W172C	516G>C	1 0.017 (0–0.065)	0 0 (0–0.004)	0 0 (0–0.010)
35delG	-	0 0 (0–0.017)	0 0 (0–0.004)	11 0.344 (0.192–0.515)
312del14	-	0 0 (0–0.017)	0 0 (0–0.004)	2 0.063 (0.007–0.172)
R75Q	224G>A	0 0 (0–0.017)	0 0 (0–0.004)	1 0.031 (0–0.118)
	IVS1+27G>C	1 0.017* (0–0.066)	-	0 0** (0–0.059)
V27I	79G>A	10 0.167 (0.085–0.271)	34 0.131 (0.093–0.175)	0 0 (0–0.010)
[V27I; E114G]	79G>A+341A>G	7 0.117 (0.049–0.210)	17 0.065 (0.039–0.099)	0 0 (0–0.010)
F191L	571T>C	0 0 (0–0.017)	1 0.004 (0–0.016)	0 0 (0–0.010)
V153I	457G >A	0 0 (0–0.017)	1 0.004 (0–0.016)	0 0 (0–0.010)

^a ^the total number of unrelated Altaian chromosomes (n = 60) is the sum of twenty-six independent Altaian patients (52 chromosomes) and eight independent patients of mixed (Altaian/other) ethnicity (8 chromosomes); ^b ^the total number of unrelated Russian chromosomes (n = 32) is estimated from thirteen independent Russian patients (26 chromosomes) plus six chromosomes corresponding to six independent patients of mixed (Russian/other) ethnicity; * 58 chromosomes were tested for the *GJB2 *non-coding exon; ** 16 chromosomes were tested for the *GJB2 *non-coding exon.
